# Effect of an intensive lifestyle intervention on the structural and functional substrate for atrial fibrillation in people with metabolic syndrome

**DOI:** 10.1093/eurjpc/zwad380

**Published:** 2023-12-15

**Authors:** Xavier Rossello, Raúl Ramallal, Dora Romaguera, Ángel M Alonso-Gómez, Alvaro Alonso, Lucas Tojal-Sierra, Carlos Fernández-Palomeque, Miguel Ángel Martínez-González, María Garrido-Uriarte, Luis López, Agnes Díaz, Olatz Zaldua-Irastorza, Amit J Shah, Jordi Salas-Salvadó, Montserrat Fitó, Estefania Toledo

**Affiliations:** Hospital Universitari Son Espases, Health Research Institute of the Balearic Islands (IdISBa), Carretera de Valldemossa, 79, 07120 Palma, Spain; Cardiology Department, Hospital Universitari Son Espases, Palma, Spain; Department of Cardiology, University Hospital of Navarra, Servicio Navarro de Salud Osasunbidea, IDISNA, Pamplona, Spain; Hospital Universitari Son Espases, Health Research Institute of the Balearic Islands (IdISBa), Carretera de Valldemossa, 79, 07120 Palma, Spain; CIBER Physiopathology of Obesity and Nutrition (CIBEROBN), Carlos III Health Institute (ISCIII), C/Monforte de Lemos, 3–5, 28029 Madrid, Spain; CIBER Physiopathology of Obesity and Nutrition (CIBEROBN), Carlos III Health Institute (ISCIII), C/Monforte de Lemos, 3–5, 28029 Madrid, Spain; Bioaraba Health Research Institute, Osakidetza Basque Health Service, Araba University Hospital, University of the Basque Country UPV/EHU, Vitoria-Gasteiz, Spain; Department of Epidemiology, Rollins School of Public Health, Emory University, Atlanta, GA, USA; CIBER Physiopathology of Obesity and Nutrition (CIBEROBN), Carlos III Health Institute (ISCIII), C/Monforte de Lemos, 3–5, 28029 Madrid, Spain; Bioaraba Health Research Institute, Osakidetza Basque Health Service, Araba University Hospital, University of the Basque Country UPV/EHU, Vitoria-Gasteiz, Spain; Hospital Universitari Son Espases, Health Research Institute of the Balearic Islands (IdISBa), Carretera de Valldemossa, 79, 07120 Palma, Spain; Cardiology Department, Hospital Universitari Son Espases, Palma, Spain; CIBER Physiopathology of Obesity and Nutrition (CIBEROBN), Carlos III Health Institute (ISCIII), C/Monforte de Lemos, 3–5, 28029 Madrid, Spain; Department of Preventive Medicine and Public Health, University of Navarra, IdiSNA, Pamplona, Spain; Department of Nutrition, Harvard T.H. Chan School of Public Health, Boston, MA, USA; Bioaraba Health Research Institute, Osakidetza Basque Health Service, Araba University Hospital, University of the Basque Country UPV/EHU, Vitoria-Gasteiz, Spain; Cardiology Service, Hospital de Manacor, Manacor, Spain; Cardiology Service, Clinica Universidad de Navarra, Pamplona, Spain; Bioaraba Health Research Institute, Osakidetza Basque Health Service, Araba University Hospital, University of the Basque Country UPV/EHU, Vitoria-Gasteiz, Spain; Department of Epidemiology, Rollins School of Public Health, Emory University, Atlanta, GA, USA; Department of Medicine, Division of Cardiology, Emory University School of Medicine, Atlanta, GA, USA; CIBER Physiopathology of Obesity and Nutrition (CIBEROBN), Carlos III Health Institute (ISCIII), C/Monforte de Lemos, 3–5, 28029 Madrid, Spain; Human Nutrition Unit, Department of Biochemistry and Biotechnology, Rovira i Virigili University, Reus, Spain; CIBER Physiopathology of Obesity and Nutrition (CIBEROBN), Carlos III Health Institute (ISCIII), C/Monforte de Lemos, 3–5, 28029 Madrid, Spain; Cardiovascular and Nutrition Research Group, Hospital del Mar Medical Research Institute (IMIM), Barcelona, Spain; CIBER Physiopathology of Obesity and Nutrition (CIBEROBN), Carlos III Health Institute (ISCIII), C/Monforte de Lemos, 3–5, 28029 Madrid, Spain; Department of Preventive Medicine and Public Health, University of Navarra, IdiSNA, Pamplona, Spain

**Keywords:** Intensive lifestyle intervention, Atrial fibrillation, Obesity, Metabolic syndrome, Mediterranean diet

## Abstract

**Aims:**

To evaluate the effect of an intensive lifestyle intervention (ILI) on the structural and functional cardiac substrate of atrial fibrillation (AF) in overweight or obese people with metabolic syndrome (Mets).

**Methods and results:**

Participants of the PREvención con DIeta MEDiterranea-Plus trial (*n* = 6874) were randomized 1:1 to an ILI programme based on an energy-reduced Mediterranean diet, increased physical activity, and cognitive-behavioural weight management or to a control intervention of low-intensity dietary advice. A core echocardiography lab evaluated left atrial (LA) strain, function, and volumes in 534 participants at baseline, 3-year, and 5-year follow-ups. Mixed models were used to evaluate the effect of the ILI on LA structure and function. In the subsample, the baseline mean age was 65 years [standard deviation (SD) 5 years], and 40% of the participants were women. The mean weight change after 5 years was −3.9 kg (SD 5.3 kg) in the ILI group and −0.3 kg (SD 5.1 kg) in the control group. Over the 5-year period, both groups experienced a worsening of LA structure and function, with increases in LA volumes and stiffness index and decreases in LA longitudinal strain, LA function index, and LA emptying fraction over time. Changes in the ILI and control groups were not significantly different for any of the primary outcomes {LA emptying fraction: −0.95% [95% confidence interval (CI) −0.93, −0.98] in the control group, −0.97% [95% CI −0.94, −1.00] in the ILI group, *P*_between groups_ = 0.80; LA longitudinal strain: 0.82% [95% CI 0.79, 0.85] in the control group, 0.85% [95% CI 0.82, 0.89] in the ILI group, *P*_between groups_ = 0.24} or any of the secondary outcomes.

**Conclusion:**

In overweight or obese people with Mets, an ILI had no impact on the underlying structural and functional LA substrate measurements associated with AF risk.

**See the editorial comment for this article ‘Lifestyle intervention on left atrium size and function in patients with metabolic syndrome', by A. Attanasio *et al.*, https://doi.org/10.1093/eurjpc/zwae048.**

## Introduction

Atrial fibrillation (AF) is the most prevalent cardiac arrhythmia and a risk factor for stroke, heart failure, dementia, and mortality.^1,2^ Atrial fibrillation is a progressive disease, with many patients advancing over time from subclinical states (changes in the atrial substrate) to clinical forms of the arrhythmia (paroxysmal, persistent, and then permanent AF).^3^ Before AF occurs, several adaptive functional and structural changes may take place in the left atrium (LA).^3^ Echocardiography is the foremost imaging modality employed to study the structure and function of the LA. Atrial structural anatomy can be evaluated using either two-dimensional (2D) or three-dimensional transthoracic echocardiography (TTE). For early detection of functional changes before anatomical changes become evident, 2D speckle-tracking echocardiography (2DSTE) is a more sensitive technique. Strain imaging provides reliable information on myocardial deformation by estimating differences in myocardial velocities. Left atrial strain is not commonly obtained, yet growing literature has supported its role in numerous outcomes.^4^ These atrial changes, as well as the likelihood to progress to AF, are mostly determined by the presence of concomitant risk factors. Whether or not risk factor control may influence pathological LA changes predisposing to AF is not known, but it is important when considering public health prevention programming.

Both metabolic syndrome (Mets) and increased body weight are major risk factors for AF development and progression since they have an impact on cardiovascular haemodynamics as well as on cardiac function and structure.^5–7^ Several studies have shown that lifestyle changes controlling such risk factors may play a role in AF prevention.^3,8^ In this context, it must be mentioned that a meta-analysis of randomized and observational studies reported that weight loss in obese individuals was associated with favourable haemodynamic effects,^9^ whereas a *post hoc* analysis of the Prevención con Dieta Mediterránea (PREDIMED) trial showed a reduced risk of newly diagnosed AF in those allocated to a nutritional intervention fostering adherence to a Mediterranean diet (MedDiet) enriched with extra-virgin olive oil in comparison with those who were allocated to the advice on a low-fat diet.^8^ Although the underlying pathophysiology mediating this effect is unknown, it seems plausible that lifestyle interventions can favourably reverse the remodelled atrial substrate or slow down its remodelling in people with increased weight and Mets and therefore reduce their natural progression to AF.^10^ Investigations of such effects are important for the validation of previous studies and personalization of therapies in at-risk individuals.

In this ancillary study of the PREDIMED-Plus trial, we aimed to evaluate the effect of an intensive lifestyle intervention (ILI) based on an energy-reduced MedDiet, increased physical activity, and cognitive-behavioural weight management on the underlying structural and functional cardiac substrate of AF in overweight or obese people with Mets. A secondary aim was to assess the association of weight loss, waist circumference reduction, increase in energy-reduced MedDiet, and increase in physical activity with changes in echocardiographic measures at 3- and 5-year follow-ups.

## Methods

### Study population

Participants were recruited for the PREDIMED-Plus (ISRCTN89898870), which is a multi-centre, randomized trial for the primary prevention of cardiovascular disease (CVD) in overweight/obese individuals with Mets. Between 2013 and 2016, 3574 men (aged 55–75 years) and 3300 women (aged 60–75 years) with a body mass index (BMI) ≥27 and <40 kg/m^2^ meeting ≥3 criteria for the Mets were recruited from 23 centres in Spain. Metabolic syndrome was defined according to the International Diabetes Federation, American Heart Association, and National Heart, Lung, and Blood Institute criteria.^11^ Participants were randomized 1:1 to an ILI programme based on an energy-reduced MedDiet, increased physical activity, and cognitive behavioural weight management or to a control intervention of low-intensity dietary advice on the MedDiet for at least 6 years. The primary endpoints of the trial are (i) a composite of non-fatal myocardial infarction, non-fatal stroke, or CVD death and (ii) weight loss and long-term weight-loss maintenance.

**Figure 1:**
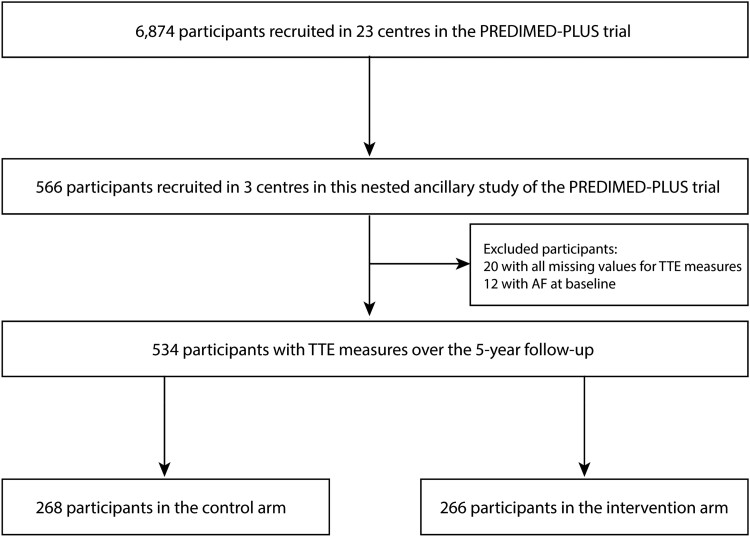
Study flow chart.

In this ancillary study of the PREDIMED-Plus trial, a subsample of 566 participants from 3 centres (University of Navarra-Preventiva, Hospital Universitario Araba, and Hospital Universitari Son Espases) were invited to evaluate the effect of the intervention on the underlying atrial substrate of AF. In this substudy, patients prospectively underwent TTE at baseline, 3-year, and 5-year follow-ups according to a uniform protocol, which has been detailed previously.^11^ After excluding participants with missing values in all TTE measures (*n* = 20) and participants with baseline AF (*n* = 12), 534 participants remained in the study population (*Figure 1*). The presence of controlled AF was not an exclusion criterion in PREDIMED-Plus, but we excluded those with prevalent AF, based on data derived from the electrocardiogram performed at recruitment. Other exclusion criteria are listed in Supplementary material online, *Table S1*.

Images were then transferred to a core lab for their assessment.^12^ The rationale and design of the corelab Palma Echo Platform has been published elsewhere.^12^ Briefly, echocardiographic examinations were performed using an ultrasound scanner Vivid 7 or Vivid 9 (General Electric Healthcare) following common procedures extensively described. M-mode, Doppler imaging, and 2D cine loops for three heartbeats of standard views were obtained from each patient. All images were digitally stored, and an offline ultrasound software EchoPAC 204 (GE Healthcare) was used for analysis. Assessment of the LA was measured by 2D 2DSTE. Four-chamber and two-chamber apical views, not foreshortened, were used. The algorithm then calculated LA strain values for the reservoir, conduit, and contractile functions, plus the maximum LA volume. An image illustrating the measurement has been added as supplementary material (see Supplementary material online, *Figure S1*).

### Study interventions

Participants randomized to the intervention group were instructed to follow an energy-reduced MedDiet, accompanied by physical activity promotion and behavioural support, with the purpose of accomplishing specific weight-loss objectives.^13^ Trained dietitians conducted a group session, an individual motivational interview, and a phone call each month during the first year. Considering the baseline customary levels of physical activity of each participant in the intervention group, a reduction of ∼30% of estimated energy requirements (a reduction of roughly 600 kcal/day) was recommended. Participants in the control group attended two educational sessions per year on the traditional MedDiet without caloric restriction, all contents previously used in the PREDIMED trial,^14^ and general lifestyle recommendations according to the standard of care. Participants were also encouraged to progressively increase aerobic physical activity (PA) to ≥150 min/week of moderate-to-vigorous PA, with the final goal of walking ≥45 min/day or equivalent and doing specific exercises to improve strength, balance, and flexibility. Compliance with PA recommendations was evaluated using validated questionnaires. Both groups of participants were supplied for free with extra-virgin olive oil (1 L/month), and all participants were encouraged to consume 500 g/month of mixed nuts.

### Study outcomes

Transthoracic echocardiography tests longitudinally and systematically assessed the structural and functional features of the LA and other cardiac chambers. Data acquisition was performed according to the standards of the European Association of Echocardiography and the American Society of Echocardiography.^15^ Transthoracic echocardiographies were performed at each site at baseline, 3-year, and 5-year follow-ups following a standardized protocol.^12^ Images were obtained at each centre by cardiologists with expertise in cardiac imaging, but they were assessed in a blind fashion by two independent expert cardiologists (core lab evaluators) not involved in obtaining the images. Each cardiologist was responsible for assessing a set of outcomes, so that each echocardiographic measure was assessed by one single cardiologist through the entire follow-up. The main study outcomes were LA emptying fraction, LA longitudinal strain—relative change in length of the tissue segment compared with its original length—, indexed and maximum LA volume, LA conduit strain—deformation in length of the LA during the passive filling phase of the cardiac cycle—, pump strain—deformation in length of the LA contraction or active emptying phase of the cardiac cycle—, LA stiffness index—as a measure of LA compliance—, and LA function index—overall performance of the LA.^16–18^

### Other variables

In addition to the imaging data, some clinical information was collected, such as age, sex, cardiovascular risk factors, and medical history at baseline. Anthropometric evaluations (weight, height, and waist circumference) were measured according to the PREDIMED-Plus protocol. Body mass index was calculated as weight (kg) divided by the square of height (m^2^).

### Statistical analysis

These statistical analyses had two separate aims. The primary aim was to assess the effect of the ILI on the structural and functional cardiac substrate of AF, using TTE tests performed at baseline, 3-year, and 5-year follow-ups. There were two primary outcomes: LA emptying fraction and LA peak systolic longitudinal strain. Additionally, there were six secondary outcomes: indexed and maximum LA volume, LA conduit and pump strain, LA stiffness index, and LA function index.^17^ These outcomes collectively allowed us to measure both intermediate-term functional effects that may be more dependent on real-time risk factor control, as well as long-term structural effects that may improve more slowly over time in response to lifestyle change.^19^

The secondary aim was to estimate the association between changes in weight, waist circumference, adherence to the energy-reduced MedDiet, and physical activity and changes in echocardiographic measures at 3- and 5-year follow-ups in the overall sample (combining both groups). This allowed us to evaluate which specific component of the ILI may be the most impactful in reducing AF risk via LA substrate changes. We dichotomized the ILI changes to provide more clinical context/interpretability. According to the PREDIMED-Plus protocol, a clinically relevant weight loss was defined as a reduction in baseline body weight of over 8% and a reduction in waist circumference of over 5%. For adherence to energy-reduced MedDiet, any improvement in the p17 questionnaire was defined as a change. For physical activity, achieving ≥150 min/week of moderate-to-vigorous exercise was defined as clinically meaningful. These changes were assessed over the first 6 months of follow-up.

Baseline characteristics were summarized as frequencies with percentages or means with standard deviation (SD) and presented by the study arm. We used mixed linear models to estimate the differences in changes in LA measures from baseline to Year 3 and to Year 5 according to the intervention group. Time was modelled with 2 degrees of freedom (accounting for between-group differences in changes between Year 3 and baseline and between Year 5 and baseline). All models were adjusted for the recruitment centre. We fitted a two-level mixed linear model with random intercepts at the cluster family and participant levels. In an additional analysis, to address the relative changes in LA measurements over follow-up, we used linear mixed models with log-transformed LA measurements to assess the changes over time according to the intervention group. Afterwards, estimates were back-transformed for an easier interpretation. As sensitivity analyses, we repeated the main analyses with multiple imputation methods using an iterative Markov chain Monte Carlo method (STATA ‘mi’ command). We generated 20 imputations for each missing measurement. Imputed missing values were used for follow-up data but not for baseline data. Missing values for each outcome at baseline and follow-up are reported in Supplementary material online, *Table S2*.^20^ To mitigate the potential impact of poor image quality, an additional analysis was conducted excluding participants with suboptimal echocardiographic windows. All analyses were conducted on an intention-to-treat basis.

To estimate the association of changes in weight, waist circumference, adherence to the energy-reduced MedDiet, and physical activity, with changes in echocardiographic measures, a separate model was used to evaluate the impact of each individual component on LA emptying fraction and LA peak systolic longitudinal strain. In these models, the independent variables were the change (difference) between baseline and the 6-month post-randomization visit in each one of the intervention components defined using the 17-item energy-reduced MedDiet questionnaire, weight in kilograms, waist circumference, and physical activity based on the goals set to be achieved after 6 months in the trial protocol. For these analyses, we used two-level mixed linear models with random intercepts at the cluster family and participant levels adjusted for the recruitment centre, the intervention group, age, sex, prevalent diabetes, use of blood pressure–lowering drugs (nine different drug families), and baseline systolic blood pressure. Ancillary, we also assessed weight, waist circumference, adherence to the energy-reduced MedDiet, and time spent doing moderate-to-vigorous physical activity over follow-up according to the intervention group, with two-level mixed linear models with random intercepts at the cluster family and participant levels adjusted for the recruitment centre.

All analyses were conducted using Stata version 16.1 (College Station, TX, USA). A *P*-value of 0.05 was considered statistically significant. GraphPad Prism version 6.00 (GraphPad Software, La Jolla, CA, USA) was used to produce the figures.

## Results

### Baseline data

The number of patients included in the analysis is summarized in the flow chart shown in *Figure 1*. Baseline characteristics of the study participants according to their randomized group are presented in *Table 1*. The mean age at inclusion was 65 years (SD 5 years), and 40% of the participants were women. Given the inclusion criteria, the prevalence of cardiovascular risk factors was high in both groups. At baseline, there were no substantial differences between groups regarding adherence to the MedDiet and total energy intake, although a higher physical activity amount was observed in the control when compared with the intervention arm. With regard to TTE baseline data, there were no major differences between groups (*Table 1*).

**Table 1:** Baseline clinical and echocardiographic data

	Control (*n* = 268)	Intervention (*n* = 266)	Overall (*n* = 534)	Total PREDIMED-Plus participants (*n* = 6874)
Baseline clinical data				
Women, %	43	37	40	49
Age, years	66 (5)	65 (5)	65 (5)	65 (5)
Body mass index, kg/m^2^	31.9 (3.2)	32.5 (3.3)	32.2 (3.3)	32.6 (3.5)
Weight, kg	85.7 (12.4)	88.5 (12.8)	87.1 (12.7)	86.6 (13.0)
Waist circumference	105 (8)	107 (8)	106 (8)	108 (10)
Smoking habit, %				
Current smoker	7	11	9	12
Former smoker	53	49	51	44
Civil status, %				
Married	79	76	78	77
Single	6	5	6	5
Widowed	7	14	10	10
Separated and divorced	7	6	6	8
Type 2 diabetes, %	29	28	28	31
Hypertension, %	88	88	88	84
Dyslipidaemia, %	74	75	74	70
Physical activity, Mets-min/day	2833 (2269)	2240 (2225)	2538 (2265)	2462 (2300)
Total energy intake, kcal/day	2467 (712)	2380 (614)	2423 (666)	2416 (633)
Adherence to the Mediterranean diet, 17-item screener	7.6 (3.0)	7.7 (2.9)	7.6 (2.9)	8.5 (2.7)
Alcohol intake, g/day	17 (21)	16 (21)	16 (21)	11 (15)
Aspirin use, %	12.3	7.9	10.1	15.6
Use of blood pressure lowering drugs, %	76.5	77.4	77.0	77.4
Use of lipid-lowering drugs, %	51.1	50.8	50.9	51.1
Insulin use, %	3.0	4.1	3.6	4.3
Metformin use, %	18.3	20.3	19.3	23.0
Baseline echo data				
Baseline LA emptying fraction, %	58.4 (9.4)	59.4 (9.4)	58.9 (9.4)	—
Baseline LA longitudinal strain, %	27.5 (6.6)	27.6 (6.5)	27.6 (6.5)	—
Indexed LA volume, mL/m^2^	22.7 (6.7)	23.1 (7.4)	22.9 (7.0)	—
LA volume, mL	43.6 (14.0)	45.0 (14.9)	44.3 (14.5)	—
Conductile LA strain, %	−11.7 (4.7)	−12.2 (4.1)	−12.0 (4.4)	—
Contractile LA strain, %	−15.7 (4.6)	−15.6 (4.6)	15.6 (4.6)	—
LA stiffness index	0.3 (0.1)	0.4 (0.2)	0.4 (0.2)	—
LA function index	67.1 (28.8)	67.4 (29.6)	67.3 (29.2)	—
LV ejection fraction, %	65.7 (7.6)	64.8 (5.8)	65.2 (6.8)	—
LV longitudinal strain	−17.7 (2.6)	−17.7 (2.4)	−17.7 (2.5)	—

LA, left atrium; LV, left ventricle.

### Treatment effect on primary and secondary outcomes

**Figure 2:**
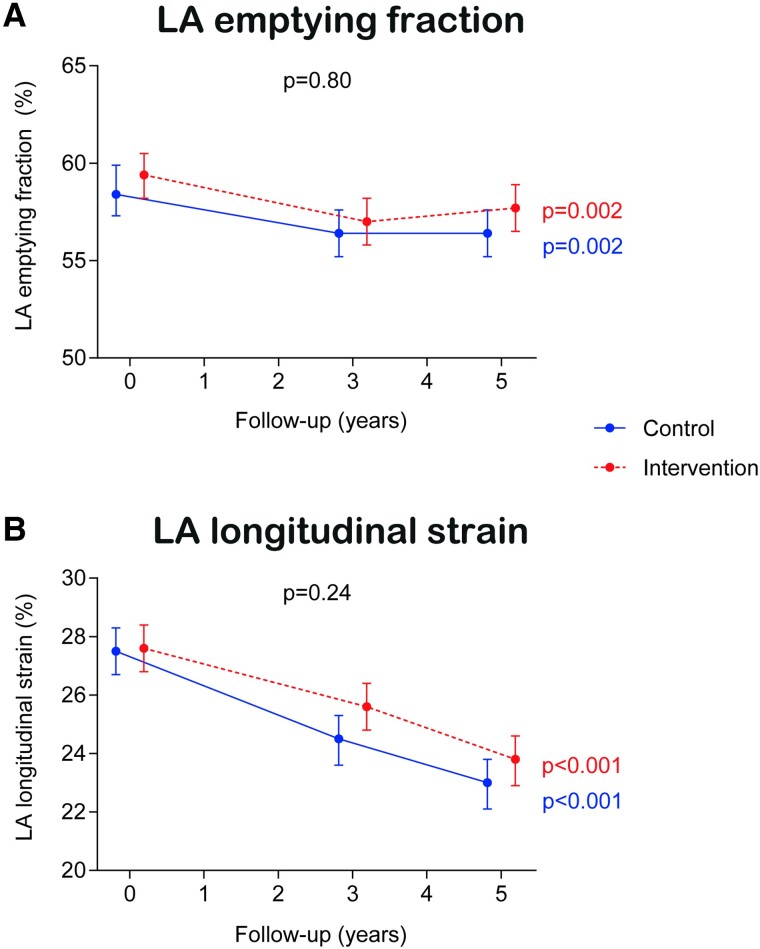
Treatment effect on co-primary outcomes. Results from linear mixed models. Time was modelled with 2 degrees of freedom. All models were adjusted for the recruitment centre. We fitted a two-level mixed linear model with random intercepts at the cluster family and participant levels.

Compared with the control group, the intervention group did not show a significant improvement in the primary outcomes (LA emptying fraction and LA longitudinal strain) over the 5-year period (*Figure 2*). Compared with baseline values, LA emptying fraction was lower at the end of the follow-up for both groups (58.4% at baseline vs. 56.4% at Year 5 for the control arm, *P* = 0.002 and 59.4% at baseline vs. 57.7% at Year 5 for the intervention arm, *P* = 0.002), but not different between them (*P*_between groups_ = 0.80). With regard to LA longitudinal strain, there was a steady decline over time in both groups (27.5% at baseline vs. 23.0% at Year 5 for the control arm, *P* < 0.001 and 27.6% at baseline vs. 23.8% at Year 5 for the intervention arm, *P* < 0.001), but similarly there was no difference between groups (*P*_between groups_ = 0.24).

**Figure 3:**
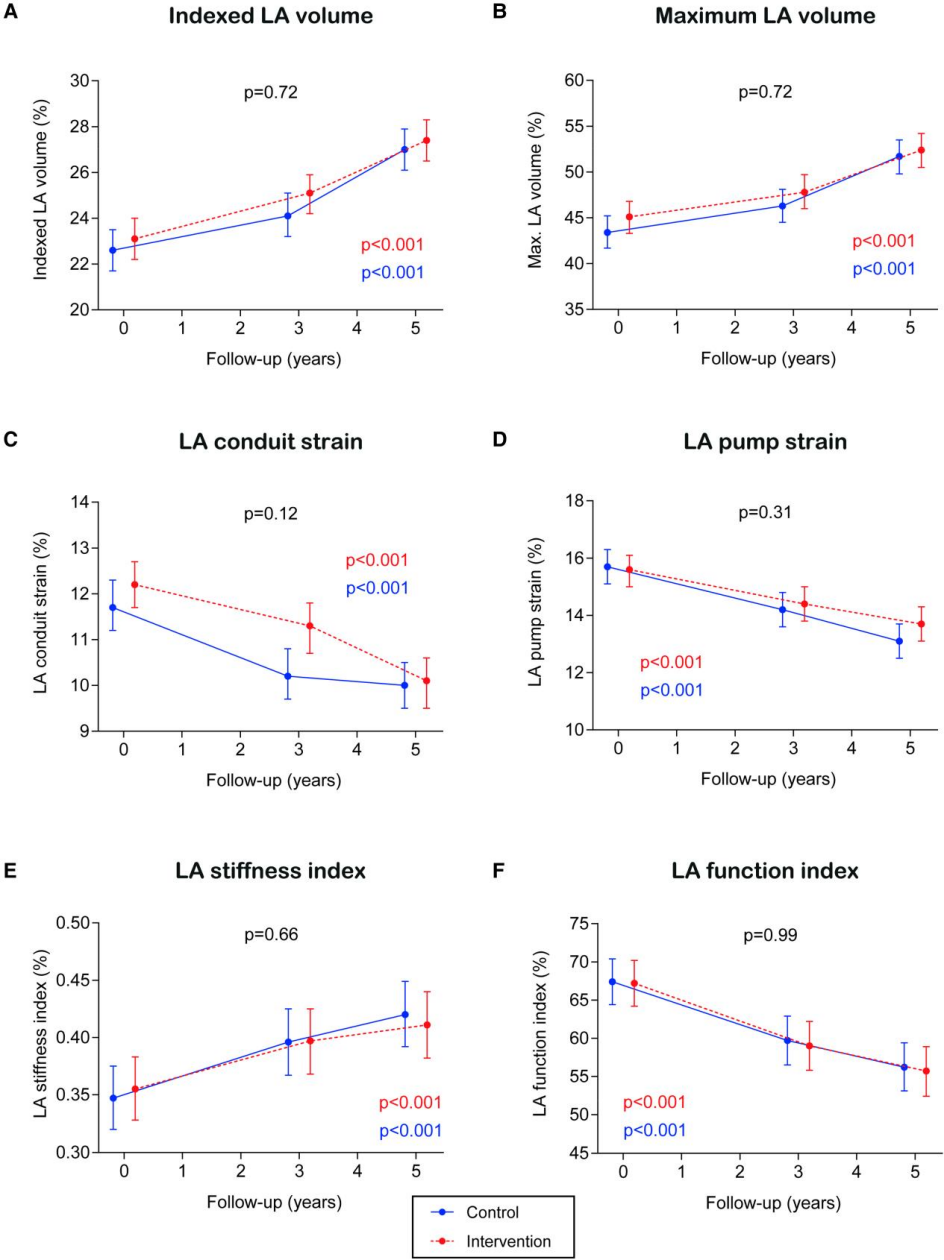
Treatment effect on secondary outcomes. Results from linear mixed models. Time was modelled with 2 degrees of freedom. All models were adjusted for the recruitment centre. We fitted a two-level mixed linear model with random intercepts at the cluster family and participant levels.

The lack of treatment effect of the intervention was also observed across all the secondary outcomes (*Figure 3*): indexed LA volume (*P*_between groups_ = 0.72), maximum LA volume (*P*_between groups_ = 0.72), LA conduit strain (*P*_between groups_ = 0.12), LA pump strain (*P*_between groups_ = 0.31), LA stiffness index (*P*_between groups_ = 0.66), and LA function index (*P*_between groups_ = 0.99). For all endpoints, changes in endpoints followed a consistent trend in the control and the intervention groups: indexed LA volume, maximum LA volume, and LA stiffness index increased over time, whereas LA conduit strain, LA pump strain, and LA function index decreased. Relative effects of the intervention on LA structural and functional echo parameters are shown as the ratios of geometric means in *Table 2*, without evidence of a statistically significant effect of the intervention on any of the echocardiographic outcomes.

**Table 2:** Relative effect of an intensive lifestyle intervention on left atrium structural and functional parameters

	Control group		ILI group		Difference ILI vs. control	
	Y3 vs. baseline	Y5 vs. baseline	Y3 vs. baseline	Y5 vs. baseline	Y3 vs. baseline	Y5 vs. baseline
LA emptying fraction	0.96 (0.94–0.99)	0.95 (0.93–0.98)	0.96 (0.93–0.98)	0.97 (0.94–1.00)	1.00 (0.96–1.03)	1.01 (0.97–1.05)
LA longitudinal strain	0.88 (0.85–0.92)	0.82 (0.79–0.85)	0.91 (0.88–0.95)	0.85 (0.82–0.89)	1.04 (0.98–1.09)	1.04 (0.98–1.10)
Indexed LA volume	1.07 (1.03–1.11)	1.19 (1.15–1.23)	1.09 (1.05–1.13)	1.18 (1.14–1.23)	1.02 (0.97–1.07)	0.99 (0.94–1.05)
LA maximum volume	1.07 (1.03–1.11)	1.19 (1.14–1.23)	1.07 (1.03–1.11)	1.16 (1.12–1.20)	1.00 (0.95–1.05)	0.98 (0.93–1.03)
LA conduit strain	0.88 (0.82–0.93)	0.84 (0.79–0.89)	0.91 (0.86–0.97)	0.82 (0.77–0.87)	1.04 (0.95–1.13)	0.98 (0.90–1.06)
LA pump strain	0.92 (0.87–0.97)	0.84 (0.79–0.89)	0.92 (0.87–0.97)	0.88 (0.83–0.93)	1.01 (0.93–1.09)	1.05 (0.97–1.13)
LA stiffness index	1.11 (1.05–1.17)	1.17 (1.11–1.23)	1.07 (1.02–1.13)	1.10 (1.04–1.16)	0.97 (0.90–1.04)	0.94 (0.87–1.02)
LA function index	0.89 (0.84–0.94)	0.82 (0.78–0.87)	0.88 (0.83–0.93)	0.82 (0.78–0.87)	0.99 (0.92–1.07)	1.00 (0.92–1.08)

Before using the mixed models, the variables were log-transformed. Later, they were back-transformed to enable easier interpretation. In this table, the values represent the ratios of geometric means, alongside their 95% CI. The models were adjusted for the recruitment centre.

LA, left atrial.

To evaluate the impact of missing values, the main analyses on primary and secondary outcomes were evaluated using a multiple imputation technique with 20 datasets (see Supplementary material online, *Table S3*). The findings for all outcomes were consistent with those yielded by the complete-case analysis.

To assess the influence of participants with suboptimal echocardiographic windows on the effects of ILI on primary outcomes, an additional analysis was conducted. The exclusion of these participants did not yield significant changes in the overall findings (see Supplementary material online, *Table S4*).

### Association between weight loss, waist circumference reduction, increase in energy-reduced MedDiet, and increase in physical activity and left atrial echo parameters

**Figure 4:**
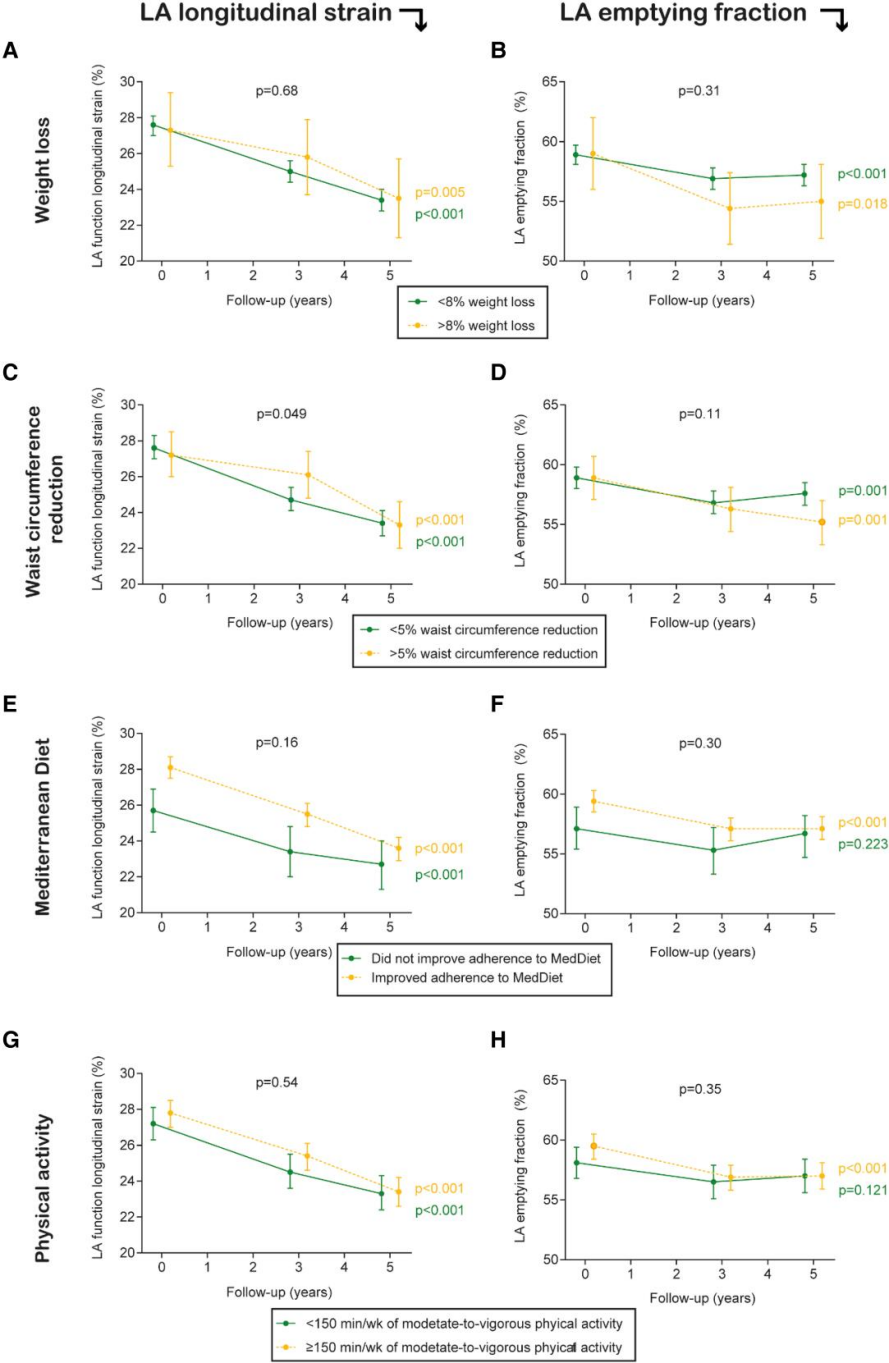
Association between weight loss, waist circumference reduction, increase in energy-reduced MedDiet, and increase in physical activity and left atrial structural and functional parameters. Results from linear mixed models. Time was modelled with 2 degrees of freedom. All models were adjusted for the recruitment centre. We fitted a two-level mixed linear model with random intercepts at the cluster family and participant levels and adjusted for the intervention group, age, sex, prevalent diabetes, use of blood pressure–lowering drugs (nine different drug families), and baseline systolic blood pressure.

The association of changes in the components of the intervention (adherence to the energy-reduced MedDiet, physical activity, weight, and waist circumference) with changes in the echocardiographic measures was evaluated in all participants (*Figure 4*).

There were no significant differences in LA longitudinal strain over a 5-year follow-up between those reducing and those not reducing >8% their weight loss (baseline vs. 5-year values for those not reducing weight loss were 27.6 vs. 23.4%, whereas they were 27.3 vs. 23.5% for those losing >8% weight; *P*_between groups_ = 0.68). There was a consistent decline in LA longitudinal strain over time in both groups (*Figure 4*A). There were significant differences in weight over follow-up between the two intervention groups (see Supplementary material online, *Figure S2*). Only participants in the ILI group significantly reduced their weight over follow-up. The mean weight change from baseline to Year 5 was −3.9 kg (SD 5.3 kg) in the ILI group and −0.3 kg (SD 5.1 kg) in the control group. Weight loss took place during the first 3 years of the intervention, and weight loss was sustained thereafter. In contrast, a borderline significant difference was observed when the change in waist circumference was evaluated (*P*_between groups_ = 0.049), though this difference was concentrated in the 3-year follow-up (*Figure 4*C). There were significant differences in waist circumference over follow-up between the two intervention groups (see Supplementary material online, *Figure S3*). Only participants in the ILI group significantly reduced their waist circumference over follow-up. The mean waist circumference change from baseline to Year 5 was −5.0 cm (SD: 8.0 cm) in the ILI group and 0.8 cm (SD: 6.3 cm) in the control group. As for weight, waist circumference reduction took place during the first 3 years of the intervention, and was sustained thereafter.

There were no significant differences in LA longitudinal strain over a 5-year follow-up between those who improved and those who did not improve the adherence to the energy-reduced MedDiet (baseline vs. 5-year values for those not improving adherence were 25.7 vs. 22.7%, whereas they were 28.1 vs. 23.6% for those improving adherence to MedDiet; *P*_between groups_ = 0.16). There was a consistent decline in LA longitudinal strain in both groups between baseline and Year 3, while in Year 5, those not improving adherence did not have as low values of LA longitudinal strain as those improving adherence (*Figure 4*E). Both groups significantly increased their adherence to the energy-reduced MedDiet, although the changes were striking for the ILI group (see Supplementary material online, *Figure S4*). The mean change in adherence to the 17-item score from baseline to Year 5 was 5.6 (SD: 4.2) in the ILI group and 4.1 (SD: 3.5) in the control group.

With regard to physical activity, there were no significant differences in LA longitudinal strain over a 5-year follow-up between those who reached and those who did not reach the goal of ≥150 min/week of moderate-to-vigorous exercises (baseline vs. 5-year values for those not reaching the goal were 27.2 vs. 23.3%, whereas they were 27.7 vs. 23.4% for those reaching that level of physical activity; *P*_between groups_ = 0.54; *Figure 4*G). There were significant differences in the time spent doing moderate-to-vigorous physical activity over follow-up between the two intervention groups (see Supplementary material online, *Figure S5*). Only participants in the ILI group significantly increased their time spent doing moderate-to-vigorous physical activity over follow-up [mean change after 5 years was 455 min/week (SD 356.7 min/week) in the ILI group and −55.6 min/week (455.0 min/week) in the control group]. This increase took place during the first 3 years of the intervention and was sustained thereafter.

The association between individual components of the intervention and LA emptying fraction was consistent with those observed for LA longitudinal strain (*Figure 4*B, D, F, and H).

## Discussion

In this nested randomized clinical trial, conducted in overweight or obese people with Mets, an ILI programme based on an energy-reduced MedDiet, increased physical activity, and cognitive behavioural weight management did not show a significant impact on the structural and functional cardiac substrate of AF compared a control intervention of low-intensity dietary advice on the MedDiet. There were significant changes in all echocardiographic endpoints (LA emptying fraction, LA peak systolic longitudinal strain, indexed and maximum LA volume, LA conduit and pump strain, LA stiffness index, and LA function index) in both arms, though these changes were similar by arm. There were no significant differences in LA longitudinal strain and LA emptying fraction based on the intervention components of weight loss, improvement in adherence to MedDiet, and physical activity. Compared with those with less waist circumference reduction, LA longitudinal strain decreased slower in those with the highest waist circumference reduction at Year 3, although this was not observed at Year 5.

People with Mets and increased body weight are at high risk for AF development and progression since these conditions have an effect on cardiovascular haemodynamics (e.g. hypertension), as well as a consequent long-term impact on cardiac function and structure.^5,6^ Some changes in LA, such as a greater increase in LA volumes and a decrease in LA function, have been considered a surrogate outcome for developing AF.^10^ Our findings do not support that an ILI plays a role in preventing LA changes, though these new results need to be put in context. Previous randomized and observational studies summarized in a meta-analysis showed that weight loss in obese individuals was associated with favourable haemodynamic effects,^9^ which can be considered the preliminary step before the changes in LA take place. A *post hoc* analysis of the PREDIMED trial showed that an intervention promoting adherence to the MedDiet enriched with extra-virgin olive oil can reduce by 40% the risk of newly diagnosed AF compared with a control intervention promoting a low-fat diet. Our data do not contradict the findings yielded by these studies, since we do not report either the haemodynamic changes or the incidence of AF over time. However, our results do not support the fact that our intervention, an ILI programme based on an energy-reduced MedDiet, increased physical activity, and cognitive behavioural weight management, has an impact on the changes that happen in LA characteristics over time in overweight or obese people with Mets, except for waist circumference at 3 years on LA strain. Visceral adiposity is likely to be one of the most important risk factors for AF, and longitudinal strain may be superior to the other measures in terms of its ability to reflect improved health status and predict AF risk.^21^ Other mechanisms, such as autonomic or electrocardiographic, warrant further examination.^22,23^

Several reasons might explain the lack of treatment effect provided by an ILI. Given that the control intervention was a dietary advice on the MedDiet, it is reasonable to think that the ‘active comparator’ might have already had an impact on LA changes in the control group.^8^ It could be that the ILI is not having an impact on LA outcomes, unlike other lifestyle interventions. This is supported by the data provided for the attainment of each individual component of the intervention. Given that those patients with a higher degree of compliance and achievements of clinically relevant goals (weight loss, waist circumference reduction, MedDiet adherence, and physical activity) did not have different outcomes than those with a lower degree of compliance, it seems that none of the individual interventions is driving any treatment effect. We tested an ILI under the hypothesis that smaller changes in LA will result in a reduced number of AF episodes in the long term. This paradigm assumes that the risk for AF is greatly dependent on the degree of LA impairment. Eventually, LA changes could be good clinical markers, but not the best surrogate endpoints. Nevertheless, in the Multi-Ethnic Study of Atherosclerosis (MESA study) study, a cohort of people free of CVD at baseline, a greater increase in LA volumes and a decrease in LA function were associated with incident AF, whereas a change in LA emptying fraction improved model discrimination and reclassification of AF risk.^10^ Finally, intervention effects on lifestyle might have attenuated over time, suggesting that sustained effects need a different strategy to change behaviours in the long term. There are several examples of a transient favourable change after a lifestyle intervention that tend to dilute over time, such as those observed in the recently published Trans-Atlantic Network to study Stepwise Non-invasive Imaging as a tool for CVD Prognosis and prevention study.^24^ Ancillary analyses conducted for this subsample showed that the lifestyle intervention led to changes in its individual components during the first 3 years of follow-up and were sustained thereafter. In addition, the COVID-19 pandemic might have had an impact on the trial conduct, though no major effects have been observed in previous reports.^25^ Our intervention has an effect on some biomarkers that are also surrogates for AF^26^ but might have little influence on echo parameters should they be already altered before enrolment in the trial (e.g. long-standing obesity).^27^ There were no significant differences in LA longitudinal strain and LA emptying fraction according to the changes in each individual component of the intervention, which was consistent with the lack of treatment effect on the primary outcomes.

Both AF and Mets are becoming a global public health issue due to the ageing of the population and the increasing levels of obesity and physical inactivity among adults in many countries.^28,29^ Although we failed to show efficacy on echocardiographic outcomes in the comparison between ILI and low-intensity dietary advice on the MedDiet, it seems plausible that other lifestyle interventions can favourably reverse the remodelled cardiac substrate of people with increased weight and Mets and therefore reduce their natural progression to AF.^10^ In the PREVEntion and regReSsive Effect of weight-loss and risk factor modification on Atrial Fibrillation study,^3^ weight loss and risk factor management reversed the type and natural progression of AF in patients with already some form of AF. Similarly, aggressive risk factor reduction improved the long-term success of AF ablation, underscoring the importance of therapy directed at the primary promoters of the AF substrate to facilitate rhythm control strategies.^30^

The use of echocardiography in this setting deserves special attention. The atrium has been historically evaluated in its morphological dimension (e.g. atrial volume). In our study, we evaluated novel parameters, such as LA strain and emptying fraction.^31,32^ Unlike traditional parameters based on blood flow during atrial contraction such as the peak A-wave velocity or the A-wave velocity time integral, LA strain represents a reliable direct measure of atrial function.^31,32^

### Strengths and limitations of this study

The strengths of this trial include an adequate sample size to fairly answer the main hypothesis, the randomized design, reducing the threat of confounding, the prolonged intervention and follow-up periods for the study of lifestyle change maintenance, the excellent retention during follow-up, and the objective measurement of the echo outcomes. Nesting this study within the PREDIMED-Plus trial provides a perfect opportunity for further comprehensive assessments of the association between lifestyle changes and myocardial derangements. Nevertheless, several limitations should be considered. This is a nationwide trial of highly selected patients (increased weight with Mets enriched with at least three factors), and therefore, the generalization of the findings should be taken cautiously.^33^ Due to the type of intervention, neither participants nor investigators were blinded to the allocation arm, although we were masked for the statistical analysis and all study outcomes relied on objective parameters not subject to self-report bias. The level of statistical significance has not been adjusted for the number of comparisons.^34^ It should be acknowledged that an optimal TTE acquisition is particularly challenging in a population with a high BMI and Mets. The obtention of standard values for LA strain in a hypothetical reference population is also challenging, given the differences between commercial softwares measuring LA strain. Other techniques, such as cardiac magnetic resonance, might be useful to complement echo measures in future studies. Finally, the long-term overall results for risk factor outcomes and other hard endpoints, such as AF or major cardiovascular events, were outside the scope of this analysis; nevertheless, significant changes in major cardiovascular risk factors after 1 year of follow-up have been observed.^13^ The results on the effect of the intervention on hard endpoints will be available after completing the follow-up for the primary outcome of the trial.

## Conclusions

Using randomized data from overweight or obese people with Mets, we observed that an ILI programme based on an energy-reduced MedDiet, increased physical activity, and cognitive-behavioural weight management did not have an effect on any of the primary (LA emptying fraction and LA peak systolic longitudinal strain) or secondary (indexed and maximum LA volume, LA conduit and pump strain, LA stiffness index, and LA function index) echocardiographic endpoints. Our findings suggest that there are age-related changes over time in these parameters that indicate increased AF risk, but that an ILI has no impact on them. Hence, an ILI had no impact on the underlying structural and functional cardiac substrate of AF in overweight or obese people with Mets. Similarly, those achieving the intervention goals in terms of weight loss, waist circumference reduction adherence to MedDiet, or physical activity showed no difference in LA longitudinal strain and LA emptying fraction compared with those with smaller clinically relevant changes.

## Supplementary material

Supplementary material is available at *European Journal of Preventive Cardiology*.

## Supplementary Material

zwad380_Supplementary_Data

## Data Availability

There are restrictions on the availability of data for the PREDIMED-Plus study, due to the signed consent agreements around data sharing, which allow access only to external researchers for research following the project purposes. Requestors wishing to access the PREDIMED-Plus trial data used in this study can place their request to the PREDIMED-Plus trial Steering Committee: predimed_plus_scommittee@googlegroups.com.
