# Supplementary material for: Effect of an intensive lifestyle intervention on the structural and functional substrate for atrial fibrillation in people with metabolic syndrome
Source: Eur J Prev Cardiol. 2023 Dec 15;31(5):629–39. doi: 10.1093/eurjpc/zwad380 (PMC10972629; doi:10.1093/eurjpc/zwad380)
Supplement: zwad380_Supplementary_Data [file zwad380_supplementary_data.docx]

# SUPPLEMENTARY MATERIAL

**Supplemental Table 1.** Exclusion criteria for the PREDIMED-PLUS trial.

| **Exclusion criteria** |
| --- |
| - Illiteracy, or inability or unwillingness to give written consent or communicate with the study staff |
| - Documented history of previous CVD, including angina; myocardial infarction; coronary revascularization procedures; stroke (either ischemic or haemorrhagic, including transient ischemic attacks); symptomatic peripheral artery disease that required surgery or was diagnosed with vascular imaging techniques; ventricular arrhythmia; uncontrolled atrial fibrillation; congestive heart failure (New York Heart Association Class II or IV); hypertrophic myocardiopathy; and history of aortic aneurism >=5.5 cm in diameter or aortic aneurism surgery |
| - Active malignant cancer or history of malignancy within the last 5 years (with exception of non-melanoma skin cancer) |
| - Impossibility to follow the recommended diet (for religious reasons, swallowing disorders, etc…) |
| - A low predicted likelihood to change dietary habits according to the Prochaska and DiClemente stages of change model. |
| - Inability to follow the scheduled intervention visits (institutionalized individuals, lack of autonomy, unable to walk, lack of a stable address, travel plans, etc.) |
| - Body weight loss > 5 kg within the prior 6 months |
| - Intention to undergo bariatric surgery |
| - History of very low-calorie diet during the prior 6 months |
| - Surgical procedures for weight loss (i.e., stomach stapling, bypass) or indication and willingness to undergo any of these procedures in the future |
| - History of small bowel resection |
| - History of inflammatory bowel disease. |
| - Obesity of known endocrine origin (with the exception of treated hypothyroidism) |
| - Food allergy to any Mediterranean diet component |
| - Immunodeficiency or HIV-positive status |
| - Cirrhosis or liver dysfunction |
| - Psychiatric disorders: schizophrenia, bipolar disease, eating disorders, or depression with hospitalization within the last 6 months |
| - Any severe co-morbidity condition with limited life expectancy |
| - Alcohol (total daily alcohol intake >50 g) or drug abuse within the past 6 months |
| - History of major organ transplantation |
| - Concurrent therapy with immunosuppressive drugs or cytotoxic agents |
| - Current treatment with systemic corticosteroids |
| - Current use of weight loss medication |
| - Concurrent participation in another randomised clinical trial |
| - Patients with an acute infection or inflammation (i.e., pneumonia) were allowed to participate in the study 3 months after resolution of their condition |
| - Any other condition that may interfere with the adherence to the study protocol |

Adapted from the PREDIMED-Plus study protocol: <https://www.predimedplus.com/wp-content/uploads/2018/11/Protocolo-PREDIMED-Plus_Eng.pdf>

**Supplementary Table 2.** Participants with missing values in the baseline and follow-up ascertainment of the primary and secondary outcomes

| Outcome | Baseline | Follow-up |
| --- | --- | --- |
| LA emptying fraction, n | 2 | 32 |
| LA longitudinal strain, n | 7 | 33 |
| Indexed maximum LA volume | 2 | 35 |
| Maximum LA volume | 2 | 32 |
| LA conduit and pump strain | 24 | 33 |
| LA stiffness index | 8 | 35 |
| LA function index | 12 | 35 |

LA, left atrial.

**Supplementary Table 3.** Sensitivity analyses of the effect of an intensive lifestyle intervention on left atrium structural and functional parameters using multiple imputations for missing data

|  | Control | Intervention | p-value  (within groups) | p-value (between groups) |  |
| --- | --- | --- | --- | --- | --- |
| LA EF |  |  |  |  |  |
| Baseline, mean (95% CI) | 58.5 (57.4-59.7) | 59.3 (58.2-60.5) | P_int_=0.002  P_con_=0.003 | 0.80 |  |
| Year 3, mean (95% CI) | 56.5 (55.2-57.7) | 57.0 (55.8-58.2) |  |  |  |
| Year 5, mean (95% CI) | 56.4 (55.2-57.7) | 57.6 (56.4-58.9) |  |  |  |
| LA longitudinal strain |  |  |  |  |  |
| Baseline, mean (95% CI) | 27.6 (26.8-28.4) | 27.6 (26.8-28.4) | P_int_<0.001  P_con_<0.001 | 0.24 |  |
| Year 3, mean (95% CI) | 24.5 (23.7-25.4) | 25.6 (24.7-26.4) |  |  |  |
| Year 5, mean (95% CI) | 23.0 (22.1-23.9) | 23.8 (22.9-24.6) |  |  |  |
| Indexed LA volume |  |  |  |  |  |
| Baseline, mean (95% CI) | 22.6 (21.7-23.4) | 23.1 (22.3-24.0) | P_int_<0.001  P_con_<0.001 | 0.72 |  |
| Year 3, mean (95% CI) | 24.1 (23.2-25.0) | 25.1 (24.2-26.0) |  |  |  |
| Year 5, mean (95% CI) | 26.9 (26.0-27.9) | 27.4 (26.5-28.4) |  |  |  |
| Maximum LA volume |  |  |  |  |  |
| Baseline, mean (95% CI) | 43.4 (41.6-45.2) | 45.2 (43.4-47.0) | P_int_<0.001  P_con_<0.001 | 0.75 |  |
| Year 3, mean (95% CI) | 46.2 (44.4-48.1) | 47.9 (46.1-49.8) |  |  |  |
| Year 5, mean (95% CI) | 51.6 (49.7-53.4) | 52.5 (50.6-54.3) |  |  |  |
| LA conduit strain | |  |  |  |  |
| Baseline, mean (95% CI) | 11.8 (11.3-12.3) | 12.1 (11.6-12.7) | P_int_<0.001  P_con_<0.001 | 0.12 |  |
| Year 3, mean (95% CI) | 10.3 (9.7-10.8) | 11.3 (10.7-11.8) |  |  |  |
| Year 5, mean (95% CI) | 10.0 (9.5-10.6) | 10.1 (9.5-10.6) |  |  |  |
| LA pump strain | |  |  |  |  |
| Baseline, mean (95% CI) | 15.7 (15.1-16.3) | 15.6 (15.0-16.2) | P_int_<0.001  P_con_<0.001 | 0.31 |  |
| Year 3, mean (95% CI) | 14.2 (13.6-14.8) | 14.4 (13.8-15.0) |  |  |  |
| Year 5, mean (95% CI) | 13.1 (12.5-13.7) | 13.7 (13.1-14.3) |  |  |  |
| LA stiffness index |  |  |  |  |  |
| Baseline, mean (95% CI) | 0.35 (0.32-0.37) | 0.36 (0.33-0.38) | P_int_<0.001  P_con_=0.001 | 0.65 |  |
| Year 3, mean (95% CI) | 0.40 (0.37-0.42) | 0.40 (0.37-0.43) |  |  |  |
| Year 5, mean (95% CI) | 0.42 (0.39-0.45) | 0.41 (0.38-0.44) |  |  |  |
| LA function index |  |  |  |  |  |
| Baseline, mean (95% CI) | 67.5 (64.4-70.5) | 67.2 (64.2-70.2) | P_int_<0.001  P_con_<0.001 | 0.96 |  |
| Year 3, mean (95% CI) | 59.9 (56.7-63.1) | 58.9 (55.7-62.0) |  |  |  |
| Year 5, mean (95% CI) | 56.3 (53.1-59.5) | 55.6 (52.4-58.9) |  |  |  |

LA, left atrial

All models were adjusted for recruitment centre.

| **Supplementary Table 4**. Relative effect of an intensive lifestyle intervention on left atrium structural and functional parameters among visits with good echocardiographic quality | | | | | | |
| --- | --- | --- | --- | --- | --- | --- |
|  | Control group | | ILI group | | Difference ILI vs. control | |
|  | Y3 vs baseline | Y5 vs baseline | Y3 vs baseline | Y5 vs baseline | Y3 vs baseline | Y5 vs baseline |
| LA emptying fraction | 0.96 (0.93-0.99) | 0.93 (0.91-0.96) | 0.96 (0.93-0.98) | 0.95 (0.93-0.98) | 1.00 (0.96-1.04) | 1.02 (0.98-1.06) |
| LA longitudinal strain | 0.92 (0.88-0.96) | 0.82 (0.79-0.86) | 0.94 (0.90-0.97) | 0.87 (0.84-0.91) | 1.02 (0.96-1.08) | 1.06 (1.00-1.12) |
| Indexed LA volume | 1.06 (1.02-1.11) | 1.22 (1.17-1.27) | 1.07 (1.03-1.11) | 1.18 (1.13-1.23) | 1.01 (0.95-1.07) | 0.97 (0.91-1.03) |
| LA maximum volume | 1.06 (1.02-1.11) | 1.21 (1.16-1.26) | 1.04 (1.00-1.09) | 1.15 (1.11-1.20) | 0.98 (0.93-1.04) | 0.95 (0.90-1.01) |
| LA conduit strain | 0.90 (0.84-0.96) | 0.84 (0.79-0.90) | 0.93 (0.87-0.99) | 0.85 (0.80-0.91) | 1.03 (0.95-1.13) | 1.02 (0.93-1.12) |
| LA pump strain | 0.96 (0.91-1.02) | 0.86 (0.81-0.92) | 0.95 (0.90-1.01) | 0.91 (0.86-0.96) | 0.99 (0.91-1.08) | 1.05 (0.97-1.14) |
| LA stiffness index | 1.07 (1.01-1.14) | 1.16 (1.09-1.23) | 1.05 (0.99-1.11) | 1.08 (1.01-1.14) | 0.98 (0.90-1.07) | 0.93 (0.85-1.01) |
| LA function index | 0.91 (0.85-0.97) | 0.79 (0.74-0.85) | 0.91 (0.85-0.97) | 0.81 (0.76-0.86) | 1.00 (0.91-1.10) | 1.02 (0.93-1.12) |
| Before performing mixed models, variables were log-transformed. Afterwards, they were back-transformed for an easier interpretation. In this table, values represent ratios of geometric means, alongside their 95% CI.  LA, left atrial  Models were adjusted for recruitment centre. | | | | | | |


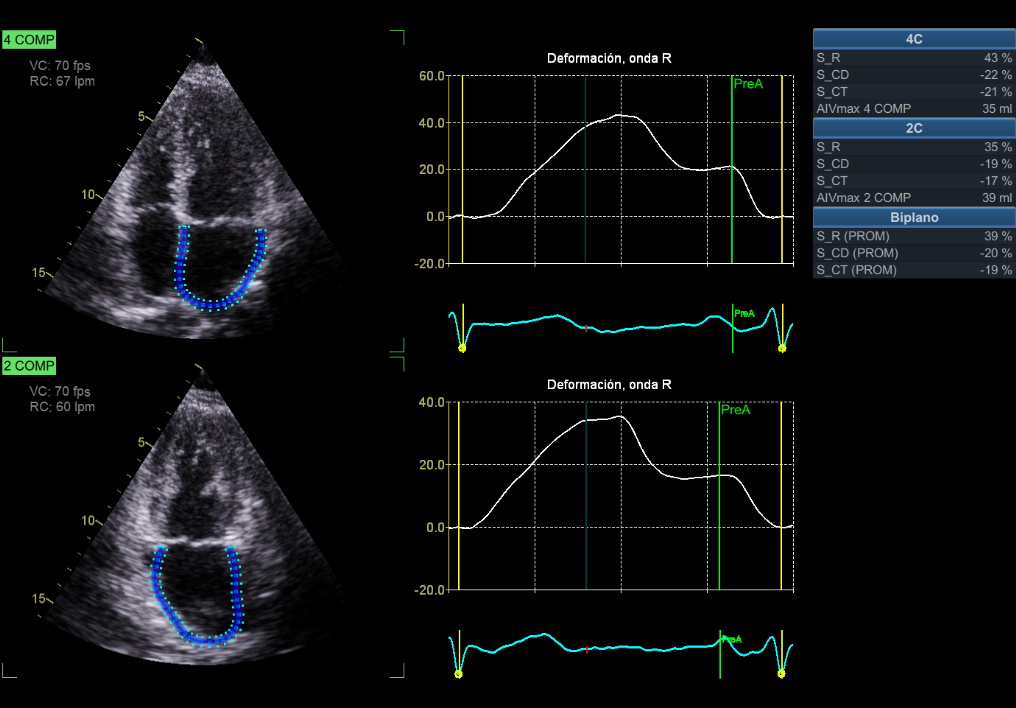


**Supplementary Figure 1**: Automatic measurement of left atrial strain imaging in the apical 4 and 2 chamber view. AIVmax: Maximal atrial volume. S_CD: peak atrial conduit strain, S_R: peak atrial contraction strain. (PROM): average.

**Supplementary figure 2.** Weight over follow-up according to the intervention group.

Results from linear mixed models. Time was modelled with 2 degrees of freedom. All models were adjusted for recruitment centre. We fitted a 2-level mixed linear model with random intercepts at cluster family and participant level.

**Supplementary figure 3.** Waist circumference over follow-up according to the intervention group

Results from linear mixed models. Time was modelled with 2 degrees of freedom. All models were adjusted for recruitment centre. We fitted a 2-level mixed linear model with random intercepts at cluster family and participant level.

**Supplementary figure 4.** Adherence to the energy-reduced Mediterranean diet over follow-up according to the intervention group.

Results from linear mixed models. Time was modelled with 2 degrees of freedom. All models were adjusted for recruitment centre. We fitted a 2-level mixed linear model with random intercepts at cluster family and participant level.

**Supplementary figure 5.** Time spent doing in moderate-to-vigorous physical activity over follow-up according to the intervention group.

Results from linear mixed models. Time was modelled with 2 degrees of freedom. All models were adjusted for recruitment centre. We fitted a 2-level mixed linear model with random intercepts at cluster family and participant level.
